# Centering and humanising health systems: empowerment through Kangaroo Mother Care

**DOI:** 10.7189/jogh.11.03105

**Published:** 2021-12-11

**Authors:** Marisa Willson, Vishwajeet Kumar, Gary L Darmstadt

**Affiliations:** 1 Stanford University, Stanford, California, USA; 2 Community Empowerment Lab, Lucknow, Uttar Pradesh. India; 3 Department of Pediatrics, Stanford University School of Medicine, Stanford, California, USA

Women often are marginalised in the care of their infants, as global health programmes tend to neglect the innate abilities women possess to enable their babies to survive and thrive. We report on the profoundly empowering experience that one of us (MW) had in providing skin-to-skin care for a preterm newborn infant in the context of programmatic efforts to transform Kangaroo Mother Care (KMC) provision in Uttar Pradesh (UP), India [1]. The Government of UP has recognised the scientific evidence and powerful humanity inherent in this practice and has constructed KMC units throughout the state with lounges designed to provide empathetic spaces in which women are dignified and rightfully recognised as possessing the ability to save newborn lives. These lounges manifest the realisation that acknowledging and unleashing human nature transforms lives.

KMC has risen to the forefront of recommended interventions to decrease neonatal mortality [2-4]. However, global uptake has been low and slow [5], neonatal deaths make up nearly half of all under-5 mortality [6], and women still do not feel empowered to take the care of themselves and their babies into their own hands. With global data now clearly indicating that KMC reduces risk of mortality of newborn infants who are under 2000 g at birth [7], there is a gaping divide between what research supports and what hospitals, governments, and societies enable women to do. In the decades of effort to decrease neonatal mortality, women have typically been circumvented in an attempt to reach the newborn with technological interventions.

## PROVIDING KMC: A PROFOUND EXPERIENCE OF EMPOWERMENT AND HEALING

**Figure 1:**
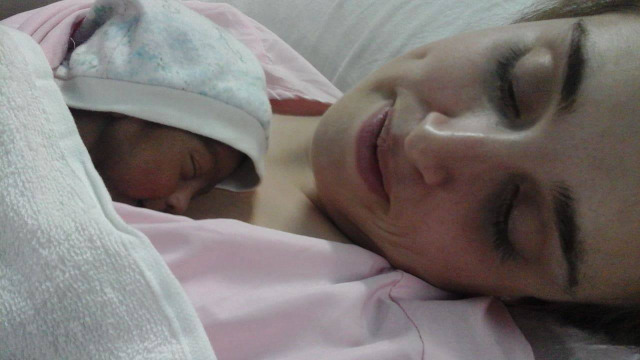
Author, Marisa Willson, providing KMC to a newborn infant at the Veerangana Avanti Bai Women’s Hospital in Lucknow.

In an effort to truly understand the power of KMC, one of us (MW) provided KMC for an infant for just one hour at the Veerangana Avanti Bai Women’s Hospital in Lucknow (**Figure 1**). This wonderfully overwhelming experience is beyond description, and here we adopt a personal (“I’) voice in an attempt to convey the impact that it had.

“When they placed the newborn baby on my chest and I felt his small heart beating against my own, I understood everything we promise to mothers when we say KMC is more than just a recommended healthcare practice. I felt love, responsibility, agency, bonding, and empowerment. I had no prior attachment to this baby; I didn’t even know his name until halfway through the session. Learning his name magnified our bonding. I wanted to stay by him, convince him I loved him, that everything would be okay, and that I would protect him. I was praying over him and singing to him. I felt that I was actually *healing* this baby as he slept on my chest, yet I realised that in the process he was also healing me. At the end of the hour, I didn’t want to give him up. I wish I had the vocabulary to adequately articulate what can only be described as the majesty of this experience. My body was doing precisely what it was built to do, a design so innate and complex that even modern technology has failed to replicate it.”

**Figure:**
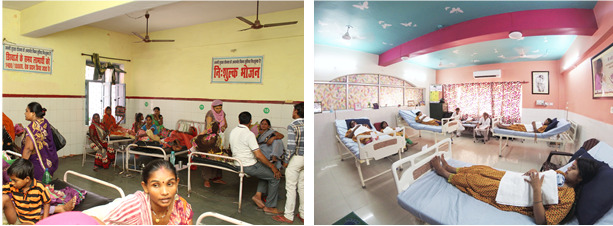
Photo: Transformation of care for newborns in Uttar Pradesh, India (left) through redesign to create KMC lounges (right) which dignify and empower women to care for their preterm infants.

## KMC: IMPETUS FOR MATERNAL AND CHILD HEALTHCARE REDESIGN

And now, we cannot help but wonder why something so beautiful, human, and impactful has only been championed by a few in the field of health care. It is with intentionality that we call this practice “innate”. Twenty-first century public health researchers did not invent KMC, they essentially gave it a name and ascribed a methodology to it. It’s proven effectiveness, however, is rooted in the undeniable power of human nature itself. Since fascination with technology has contributed to, yet has had bounded impact in curbing neonatal mortality [1], it is time to look within for solutions that engage the power of our humanity. Modern medicine must stop circumventing humanity in trying to solve public health problems.

In 2003, the team at the Community Empowerment Laboratory started a movement in UP based on the simple and powerful idea that mothers and babies should be at the center of innovation in maternal and newborn health. Fifteen years later, KMC is on the verge of an explosion in uptake through Government programming. There are now KMC units in over 170 hospitals with lounges designed to bring mothers and their preterm infants together at the center of health care. This doesn’t just heal the babies and their mothers (and potentially other caregivers); allowing ourselves to be inspired into action by this focus on humanity can also help to heal the brokenness in the health care system.

In the context of health care systems in high-income countries, there has never been a better time to redefine care delivery in a period fraught with unexpected challenges and systemic shortcomings. The COVID-19 pandemic and subsequent response has highlighted the need for medical movements larger than any one leader or technology. We have seen the devastation brought on by just one disease as preventable deaths [8] continue to mount [9]. Even under the best of circumstances, birth in the US is nowhere near as safe as it should be, especially for marginalised populations, nor as safe as we often mistakenly think it is. The US has the third worst maternal and neonatal outcomes among developed nations, falling from #12 in the 1960s to #32 in the 2010s [10]. During this unprecedented season of health care challenge and strain on our system’s resources, we suggest that it is time we look to the women bearing the brunt of these poor outcomes as pivotal to solving our shortcomings. We call on all physicians and health care professionals to reimagine the way care is delivered to women and their infants, asking that women be returned their agency in providing for themselves and their families. Too long have we ignored women, especially poor women of color, and kept them apart needlessly from their newborn infants, as demonstrated recently in a trial of immediate KMC initiated before medical stabilisation of very low birth weight infants which showed that immediate KMC reduced risk of mortality by 25% compared to highly technical incubator care [7]. Times are tough and tensions are high, but we cannot afford to lose this opportunity to redefine care when the system is at its most impacted, its most vulnerable. The future of health care delivery for mothers and newborns must involve a redesign of policies, structures, technologies, communications and ultimately power dynamics to enable mothers and newborns to be together and to heal one another, together. Empowering women to provide KMC for their newborns will lead to better health outcomes, and centering women in their own health care will lead to better societies.

We have published studies and statistics in an effort to demonstrate the benefits of this practice for newborn infants [2-5,7] as well as for mothers [11] and fathers [12] and its feasibility for scaling up [1,13]. But for anyone still skeptical, we recommend they try it. Give KMC, lay skin-to-skin with an infant, and put what research tells us into the context of this indescribable completion of your human wholeness; the proof of its power will come from within. Take an hour, feel empowered.
